# Proposal of a method for clinical validation of ICNP^®^ terminological subsets in hospital practice

**DOI:** 10.1590/0034-7167-2025-0492

**Published:** 2026-07-31

**Authors:** Richardson Augusto Rosendo da Silva, Harlon França de Menezes

**Affiliations:** I Universidade Federal do Rio Grande do Norte. Natal, Rio Grande do Norte, Brazil

**Keywords:** Nursing Process, Methods, Standardized Nursing Terminology, Nursing Diagnosis, Clinical Nursing Research., Proceso de Enfermería, Métodos, Terminología Normalizada de Enfermería, Diagnóstico de Enfermería, Investigación en Enfermería Clínica.

## Abstract

**Objectives:**

to describe a methodological proposal for the clinical validation of terminological subsets of the International Classification for Nursing Practice (ICNP^®^), applicable to hospital settings.

**Methods:**

this is a descriptive study that proposes methodological strategies for clinical validation of subsets, incorporating practical application in real care scenarios, evaluation by clinical judges, and statistical tests of agreement.

**Results:**

the method consists of five stages: (1) Application of the Nursing Process, focusing on the systematic collection of clinical data; (2) Construction of clinical case studies; (3) Clinical validation of ICNP^®^ statements, conducted by nurse clinical judges; (4) Assessment of agreement among judges using robust statistical analyses; and (5) Synthesis of findings and refinement of the terminological subset.

**Conclusions:**

the method can be replicated in different clinical contexts, strengthening the scientific basis and visibility of nursing practice.

## RESUMEN

**Objetivos:**

describir una propuesta metodológica para la validación clínica de subconjuntos terminológicos de la Clasificación Internacional para la Práctica de Enfermería (CIPE^®^), aplicable a escenarios hospitalarios.

**Métodos:**

se trata de un estudio descriptivo que propone estrategias metodológicas para la validación clínica de subconjuntos, incorporando aplicación práctica en escenarios reales de cuidado, evaluación por jueces clínicos y pruebas estadísticas de concordancia.

**Resultados:**

el método está compuesto por cinco etapas: (1) Aplicación del Proceso de Enfermería, con énfasis en la recolección sistematizada de datos clínicos; (2) Construcción de estudios de caso clínico; (3) Validación clínica de los enunciados de la CIPE^®^, realizada por jueces clínicos enfermeros; (4) Evaluación de la concordancia entre jueces mediante estadística robusta; y (5) Síntesis de los hallazgos y refinamiento del subconjunto terminológico.

**Conclusiones:**

el método puede ser replicado en diferentes contextos clínicos, fortaleciendo la cientificidad y la visibilidad de la práctica de enfermería.

## INTRODUCTION

The consolidation of the Nursing Process (NP) as a scientific method guiding clinical nursing practice requires the adoption of a standardized language capable of clearly expressing nurses’ clinical judgments, expected outcomes, and implemented interventions. In this context, the International Classification for Nursing Practice (ICNP^®^) has emerged as an enumerative, combinatorial, and globally recognized terminology designed to support the documentation, communication, and analysis of nursing care across diverse healthcare settings^(1)^.

The ICNP^®^ enables the development of terminological subsets tailored to specific populations, thereby enhancing the precision and applicability of the NP in concrete clinical situations^(2,3)^. These subsets function as strategic and technological resources that strengthen NP documentation by providing diagnoses, interventions, and outcomes aligned with standardized language and evidence-based practice. The International Council of Nurses recommends the creation of terminological subsets adapted to local needs and different care realities^(4)^.

However, the clinical validation stage, responsible for verifying the applicability of subsets in real care contexts, remains underexplored in national scientific production, limiting their effective incorporation into clinical routines and health information systems^(5,6)^.

A recent scoping review reinforced clinical validation as an essential step for consolidating ICNP^®^ terminological subsets in nursing practice. The study analyzed 15 publications and highlighted a lack of methodological uniformity, predominance of case studies, and geographic concentration of research. It also emphasized the absence of clinical validation as a structuring phase of the NP, underscoring the need for more robust guidelines. These findings support the view that clinical validation should occupy a central position, ensuring applicability, scientific rigor, and impact on the quality of healthcare delivery^(6)^.

This gap points to the urgent need for innovative, rigorous, and replicable methodologies capable of assessing, based on real clinical situations, the relevance of proposed nursing statements. Clinical validation not only strengthens the safety and accuracy of nursing decisions but also contributes to the epistemological advancement of standardized professional language.

The methodology proposed in this study was developed by the leader of the Laboratory for Research in Health and Nursing Care for People in Acute and Chronic Conditions (LAPAC), Department of Nursing, Universidade Federal do Rio Grande do Norte (UFRN). The group is certified and registered in the Directory of Research Groups (DGP) of the CNPq Lattes Platform and has over a decade of experience in implementing and clinically validating ICNP^®^ in different care settings.

Grounded in a consolidated theoretical-practical trajectory, the proposal resulted from a critical analysis of theses, dissertations, and scientific articles addressing methodological strategies for the construction and validation of ICNP^®^ terminological subsets. This process enabled the group to systematize original, coherent, and replicable steps.

This methodological structure reflects the technical and scientific maturity of LAPAC researchers in applying the NP, conducting clinical validations with expert judges, and employing robust statistical tools. Consequently, it ensures conceptual rigor, practical applicability, and adherence to normative guidelines such as ISO 18104 and COFEN Resolution No. 736/2024^(5,6)^.

Accordingly, this study proposes an innovative methodology for the clinical validation of ICNP^®^ terminological subsets, grounded in the application of the NP in real situations, the critical analysis of clinical judges, and the use of multiple statistical tests to ensure robustness and applicability of results.

The study presents a technological innovation applied to the clinical validation of ICNP^®^ terminological subsets, structured as an unprecedented and organized proposal for the practical use of standardized nursing language.

The methodology developed is distinguished by the operationalization of the NP in real clinical environments, using structured records and direct observation of clinical cases to verify the applicability of diagnostic statements, expected outcomes, and nursing interventions contained in terminological subsets previously constructed and validated by experts.

## OBJECTIVES

To describe a methodological proposal for the clinical validation of terminological subsets of the International Classification for Nursing Practice (ICNP^®^), applicable to hospital settings.

## METHODS

### Ethical Aspects

This descriptive study did not involve human participants in its execution and, therefore, did not require approval by a Research Ethics Committee.

### Descriptive Trajectory

This is a descriptive study developed according to the Standards for Quality Improvement Reporting Excellence (SQUIRE 2.0) guidelines, used as a reference to ensure standardization and quality in reports of research aimed at improving clinical practice. In this way, scientific rigor, narrative clarity, and methodological traceability are guaranteed throughout all stages of the investigation, in accordance with the items established in SQUIRE.

Initially, the study presents a detailed description of the problem and its clinical relevance, contextualizing the need to qualify the NP through the clinical validation of terminological subsets, in line with items 3 and 4 of the checklist. The theoretical foundation and rationale of the intervention are explained based on ICNP^®^ conceptual models and consolidated evidence, justifying methodological choices and supporting the effectiveness of the proposed intervention (item 5).

The objectives are presented clearly and measurably (item 6). The method describes the institutional context of the research, the characteristics of the services, the population profile, and the available resources, addressing item 7. The intervention is detailed, including the application of NP stages, the development of clinical case studies, the identification of nursing diagnoses, outcomes, and interventions, validation by clinical judges, and subsequent statistical analysis, ensuring reproducibility (item 8).

The study of the intervention and its analysis are described precisely, highlighting the approach adopted to determine whether the observed effects derived from the implemented activities, in accordance with item 9. The measures used are presented with operational definitions, validity, and reliability, as required by item 10, together with the quantitative analytical strategy (item 11).

The manuscript also addresses ethical aspects, including institutional authorization and compliance with applicable principles (item 12). The results are organized sequentially, presenting the evolution of the intervention, its effects, interactions with the context, and emerging findings (item 13). The discussion articulates the results with the initial rationale and with national and international literature, compares findings with other studies, analyzes clinical implications, and acknowledges methodological limitations (items 14, 15, and 16).

Finally, the conclusions emphasize the usefulness of the intervention, its future applicability, the potential for replication in other care contexts, and recommendations for subsequent studies, fully addressing item 17. Thus, the manuscript demonstrates strong adherence to SQUIRE 2.0 and provides a consistent methodological contribution to the improvement of clinical nursing practice.

The method consists of five stages:

1. Application of the Nursing Process, focusing on the systematic collection of data through clinical examination, interviews, validated scales, and complementary tests;
2. Construction of real clinical case studies, based on patient records, containing detailed and updated information on history, clinical condition, and care evolution;
3. Clinical validation of ICNP^®^ statements, conducted by nurse clinical judges with experience in the care area under investigation, who analyze the relevance of proposed diagnoses, outcomes, and interventions based on direct observation and documentary analysis of cases;
4. Assessment of agreement among judges, using robust statistics such as the Kappa coefficient and the Intraclass Correlation Coefficient (ICC) to verify the consistency of clinical decisions;
5. Statistical analysis and synthesis of findings, aimed at identifying clinically validated statements based on frequency, relevance, and agreement among evaluators.

These stages are described in detail in the Results section. It is noteworthy that this proposal shows greater adherence to the hospital context, whose dynamics of NP operationalization differ substantially from those observed in Primary Health Care (PHC).

## RESULTS

### Stage 1: Application of the Nursing Process with a focus on the systematic collection of clinical data

In this stage, the NP is applied to real patients in the selected clinical setting. Data collection must be conducted by specialist nurses who meet the following criteria: consistent practical experience in the area, participation in research groups or technical-scientific committees, academic production related to the topic (articles, book chapters, or conference papers), and regular involvement in scientific events and professional development activities. These requirements ensure that the specialist possesses in-depth knowledge and critical analysis skills to contribute meaningfully to the study.

The nurse may employ multiple strategies, such as patient and/or family interviews, physical examination, application of validated scales, analysis of laboratory tests and vital signs, in addition to the use of the ICNP^®^ Terminological Subset previously validated for content. Data must be organized to enable subsequent clinical judgment. As a methodological suggestion, nursing diagnoses, outcomes, and interventions identified in patients may be entered into Microsoft Office Excel^®^ spreadsheets, in exclusive tabs corresponding to each patient. These spreadsheets are then sent for validation by specialist nurses, who may include additional information regarding socioeconomic and clinical data, as well as relevant observations for the diagnostic inference process. Each specialist independently judges the presence or absence of statements in each spreadsheet.

Several ICNP^®^ terminological subsets already provide specific data collection instruments developed by their authors, such as those focused on patients with chronic kidney disease, neonatology, women’s health, and palliative care. Whenever possible, the use of these original instruments is recommended, as they are constructed based on clinical evidence and validated by area specialists, thereby strengthening methodological coherence and applicability of statements in the care context.

At this stage, it is advisable to ensure ethical consent and standardization of data sources, recording information clearly and in technical language, including disease progression, previous interventions, and treatment response.

Data collection is the initial and structuring stage of the NP, as defined by COFEN Resolution No. 736/2024^(7)^, which establishes five interdependent phases: Assessment, Diagnosis, Planning, Implementation, and Nursing Evolution. For this collection to be effective and clinically meaningful, it is essential that the instrument used be based on the subset to be validated and on a theoretical framework serving as a conceptual reference.

In addition to the ICNP^®^ Terminological Subset, the data collection instrument may be developed based on different nursing theories, which provide support for understanding the human being in its entirety and guiding care. The choice of theory must be aligned with the target population, care context, and study objectives. Regardless of the theory adopted, the instrument must include elements organized to support the Nursing Assessment stage, such as:

- Identification data and social context;
- Health history and current clinical conditions;
- Observed signs and symptoms;
- Emotional, cultural, and spiritual aspects;
- Functional capacities and limitations;
- Support network and physical environment.

These data must be categorized according to the central concepts of the chosen theory and the terminological subset, enabling systematic and evidence-based analysis. This guided collection allows for:

- Supporting clinical judgment for the identification of nursing diagnoses from the subset or formulation of new ones;
- Defining interventions consistent with identified needs or developing new ones;
- Listing expected outcomes based on theoretical and clinical indicators, including the possibility of proposing additional outcomes not present in the original subset.

The development of new statements must follow the standards of ISO 18104, which defines the structure of terminological representations in nursing, and be compatible with ICNP^®^, currently integrated into the SNOMED-CT ontology in health information systems. This guideline ensures semantic interoperability, terminological consistency, and integration with electronic health records and digital health technologies^(8)^.

It is strongly recommended that the authors of the terminological subset participate in the clinical validation process, even when the material is already published and available in open access. This is because certain conceptual, methodological, and decision-making aspects, such as criteria for term inclusion, structuring of statements, and delimitation of clinical contexts, are often not explicitly detailed in original publications and can only be understood through direct reports from the authors. Such dialogue contributes to ensuring the fidelity of subset application and coherence between the initial proposal and the validation process.

### Stage 2: Construction of Clinical Case Studies

The development of clinical case studies is based on concepts already proposed in previous studies^(6)^ and on the attributes of ISO 18104:2023, which defines the structure and elements required to represent statements in terminology systems. This standard guides the identification of key concepts, their relationships, and clinical contexts, providing an appropriate theoretical foundation for composing a minimum dataset in case study research. In addition, the construction of clinical case studies considers the core concepts of the Nursing Process (NP), as established by COFEN Resolution No. 736/2024, and the axes of the ICNP^®^, ensuring terminological coherence and practical applicability of the statements under validation.

Based on the clinical data collected, a detailed case study is prepared, representing the patient’s situation at a specific point during hospitalization or care. This study must include: demographic data; health history; current clinical conditions; relevant signs and symptoms; ongoing therapy and clinical evolution; nursing diagnoses/outcomes and interventions from the ICNP^®^ subset; as well as the inclusion of new statements identified as necessary. The objective is to transform fragmented information into an integrative clinical case that enables the clinical judge to analyze the situation holistically.

To ensure anonymity and confidentiality, clinical cases will be presented in a de-identified format, without data that allow direct or indirect identification of individuals. Each case will be identified only by a sequential number (e.g., Case 1, Case 2, Case 3), with names and initials replaced by numeric codes, in accordance with national and international ethical recommendations for research involving clinical data.

Each case study will be independently evaluated by nurse judges. A second nurse will conduct cross-validation, verifying consistency between the data presented, clinical judgment, and the relevance of the applied ICNP^®^ statements. In cases of disagreement, a consensus method will be adopted, through structured discussion among judges, until a minimum agreement of 80% is reached, in line with recommendations for content validation and inter-rater reliability^(9)^.

Each case study will be independently evaluated by nurse judges. A second nurse will conduct cross-validation, verifying consistency between the data presented, clinical judgment, and the relevance of the applied ICNP^®^ statements. In cases of disagreement, a consensus method will be adopted, through structured discussion among judges, until a minimum agreement of 80% is reached, in line with recommendations for content validation and inter-rater reliability.

The construction of clinical case studies constitutes an essential stage in the methodology for validating ICNP^®^ terminological subsets, as it enables the assessment of the applicability of statements in real care situations. According to COFEN Resolution No. 736/2024, the NP must be structured in five interrelated, cyclical stages, grounded in theoretical support and scientific evidence.

The following framework is suggested for the development of clinical case studies:

1. Definition of target population and care context: identify the population (e.g., patients with chronic kidney disease, older adults in social vulnerability); select the clinical setting (primary care, hospital, outpatient clinic); ensure alignment with the ICNP^®^ subset to be validated.
2. Collection of clinical information: describe clinical data through medical records, interviews, and physical examination, preparing a complete case with history, signs and symptoms, tests, treatments, and social context.
3. Organization according to NP stages:
  - Nursing Assessment: subjective data (interview) and objective data (physical examination), including results from scales and laboratory tests;
  - Nursing Diagnosis: clinical judgment of identified needs, using standardized ICNP^®^ language;
  - Nursing Planning: definition of priorities, expected outcomes, and prescription of interventions;
  - Nursing Implementation: description of planned actions, respecting technical competencies and collaborating with the multidisciplinary team;
  - Nursing Evaluation: assessment of achieved outcomes, revision of the plan, and systematic documentation in the medical record.
4. Insertion of ICNP^®^ statements: select diagnoses, outcomes, and interventions from the original subset; include new statements identified as necessary; relate them to case data, justifying their clinical relevance.

### Stage 3: Clinical Validation of ICNP® Statements by Clinical Judges

In this stage, nurses with practical experience in the thematic area (e.g., oncology, women’s health, intensive care, primary care, among others) act as clinical judges. The case studies are presented along with the ICNP^®^ subset statements previously selected.

Each judge evaluates the relevance of the statements in relation to the clinical case using a Likert-type scale ranging from 1 to 4, where 1 = not relevant, 2 = slightly relevant, 3 = relevant, and 4 = highly relevant. Judges may also provide qualitative comments. The use of this scale is recommended as it allows for a more refined assessment of the degree of agreement among judges, reducing dichotomization of responses and enabling more robust statistical analyses of concordance. Other evaluation criteria may also be incorporated at this stage, such as clarity, applicability, and relevance. Semantic adjustments are usually made during the content validation stage; if necessary, authors may implement adjustments in new versions of the classification.

The following criteria are recommended for the selection of clinical judges: professionals must work in the specific area of interest of the subset, hold at least a specialist degree or have proven experience, demonstrate availability for the validation stage, and show familiarity with ICNP^®^. In addition, it is advisable to incorporate criteria specifically aimed at analyzing clinical applicability, including prior experience in operationalizing the NP with ICNP^®^, participation in committees or research groups related to the theme, and a minimum of two years of clinical practice with the target population or health condition under study ^(10)^.

These parameters strengthen methodological rigor and ensure that the selected specialists possess sufficient theoretical and practical competencies to evaluate the relevance and applicability of the statements.

It is recommended to use a validation tool such as a physical or digital form containing the statements organized by axis (focus, judgment, action, means, location, time, client), accompanied by instructions for clinical judgment.

To ensure validity and reliability of results, sample size calculation should be based on the expected proportion of agreement among judges, using specific formulas for inter-rater concordance studies. In general, for the Content Validity Index (CVI), a minimum of 5 to 10 judges per thematic area is suggested, while for tests such as Fleiss’ Kappa or ICC, the number of items and desired precision level should guide sample size determination.

The data collection period may vary depending on the number of clinical cases and the availability of judges; however, a predefined schedule is recommended, with collection, analysis, and validation stages distributed over 26 weeks^(6)^.

Regarding data collectors’ training, it is essential that nurses responsible for data collection receive specific training on the use of the instrument, clinical recording criteria, ICNP^®^ foundations, and NP stages, ensuring standardization, quality, and reliability of the information obtained.

### Stage 4: Statistical Evaluation of Agreement Among Judges

In this stage, the clinical judgments made by nurse evaluators regarding the relevance of ICNP^®^ diagnostic, outcome, and intervention statements are organized into spreadsheets and subjected to statistical analysis. The objective is to identify the degree of consensus and/or agreement among judges, as well as to verify the statistical significance of variations observed in their responses. For this purpose, the Content Validity Index (CVI) is used, considering statements validated when CVI ≥ 0.80.

Although CVI is widely employed in validation studies, particularly in health research, its use has been criticized by psychometric researchers. The main limitations include the absence of statistical precision measures, subjectivity in defining cutoff points (such as the traditional ≥ 0.80), and the inability to capture nuances in agreement among evaluators. Furthermore, CVI does not account for chance, which may overestimate item validity in contexts with a reduced number of judges.

Given these limitations, it is also recommended to calculate the Kappa coefficient and/or the Intraclass Correlation Coefficient (ICC), which allow for more robust measurement of agreement among multiple judges. Additional tests may also be applied to verify statistical differences among evaluations. Recommended tools for such analyses include Excel, SPSS, R, and online software with specific concordance modules.

Therefore, in studies aiming to propose methodologies for clinical validation, it is essential that the statistical tests employed are aligned with the type of data, the number of evaluators, and the objective of the analysis. In this regard, the use of Tables 1 and 2 in this study contributes to methodological coherence by offering a repertoire of applicable tests and their respective statistical hypotheses, thereby strengthening scientific rigor and the validity of results.

The methodological proposal outlined seeks to ensure coherence between the objectives of clinical validation of ICNP^®^ terminological subsets and the statistical procedures adopted. To this end, Charts 1 and 2 were designed to systematically guide the choice of the most appropriate statistical tests according to data type, number of judges, and specific analysis objectives.

**Chart 1:** Comparison of statistical tests applicable to the clinical validation of ICNP^®^ terminological subsets, Natal, Rio Grande do Norte, Brazil, 2026

Statistical Test	What does it assess?	When to use?	Practical Example	Interpretation
CVI (Content Validity Index)	Proportion of judges who consider the item relevant	When responses are binary (yes/no)	12 out of 15 judges validate a diagnosis → CVI = 0.80	CVI ≥ 0.80 indicates that the item is clinically valid
Fleiss’ Kappa	Degree of agreement beyond chance among 3 or more judges	When ≥ 3 judges assess item relevance (yes/no)	Three groups of judges evaluate 25 diagnoses	>0.80: excellent agreement; <0.40: poor agreement
ICC (Intraclass Correlation Coefficient)	Consistency of scores assigned among judges	When using an ordinal scale (e.g., Likert) with ≥ 2 judges	Judges evaluate 10 interventions on a scale from 1 to 4	ICC > 0.75: good agreement; ICC > 0.90: excellent agreement
Kendall’s W	Degree of agreement in item ranking by judges	When judges rank/order items by importance	Judges rank 8 interventions from most to least appropriate	W close to 1: high agreement; W < 0.5: low agreement
Binomial Test	Whether the proportion of “yes” differs from a theoretical proportion	To verify if the expected proportion of responses (e.g., 80%) is achieved	Expected 80% agreement, but only 9/15 judges validate the item	p < 0.05 indicates a significant difference from the expected proportion
Kruskal-Wallis Test	Difference between medians of ≥ 3 independent groups	When judges come from different areas or profiles	Judges from different care settings evaluate the relevance of a set of diagnoses	p < 0.05: at least one group differs; use post-hoc (Dunn) to locate the difference
Friedman Test	Difference in repeated evaluations of ≥ 3 conditions by the same judges	When the same judges evaluate multiple items on an ordinal scale	Five judges evaluate 6 diagnoses on a scale from 1 to 4	p < 0.05: significant difference among items; post-hoc Dunn test may be applied
Repeated Measures ANOVA	Difference in means of evaluations by the same judges	When ≥ 3 evaluations of the same group with parametric data are available	Same judges evaluate 4 diagnoses at different time points	p < 0.05: difference among means; post-hoc (Tukey or Bonferroni) identifies where
Wilcoxon Test	Difference between two paired evaluations (non-parametric)	When the same judges evaluate items in two rounds	Comparison between evaluation 1 and 2 after term adjustment	p < 0.05: significant change between evaluations
Paired t-test	Difference between means of two paired evaluations with normal data	When two rounds of evaluation have normal distribution	Same judges evaluate 20 terms before and after revision	p < 0.05: means are significantly different between the two time points

**Chart 2:** Statistical hypotheses appropriate for each test applied to the clinical validation of ICNP^®^ terminological subsets, Natal, Rio Grande Norte, Brazil, 2026

Test	H₀ (Null Hypothesis)	H₁ (Alternative Hypothesis)
CVI (Content Validity Index)	CVI ≤ 0.80 does not validate the item	CVI ≥ 0.80 validates the item
Fleiss’ Kappa	Agreement among judges is equal to chance	Agreement is greater than chance
ICC (Intraclass Correlation Coefficient)	No consistency among judges (ICC ≈ 0)	Statistically significant consistency among judges (ICC > 0)
Kendall’s W	No agreement among judges (W ≈ 0)	Significant agreement among judges (W > 0)
Kruskal-Wallis	Group medians are equal	At least one median is different
Repeated Measures ANOVA	Evaluation means are equal	At least one mean is different
Friedman Test	Evaluation distributions are equal	At least one distribution is different
Wilcoxon Test	Distributions between rounds are equal	Distributions are different
Paired t-test	Means are equal between two rounds	Means are different
Binomial Test	Observed proportion = expected proportion (e.g., 0.80)	Observed proportion ≠ expected proportion

Table 1 presents a comparison of different concordance and validity tests, highlighting their applications, practical examples, and interpretation criteria. Table 2 specifies the statistical hypotheses (null and alternative) associated with each test, reinforcing the theoretical and methodological foundation of the analysis.

Thus, the use of these tables ensures alignment between the nature of the clinical validation study and the statistical methods employed, promoting greater rigor, transparency, and reproducibility of results.

Chart 2 presents the null and alternative hypotheses appropriate for each test applied to the clinical validation of ICNP^®^ terminological subsets.

### Stage 5: Synthesis of Findings and Refinement of Terminological Subsets

Statements that demonstrated high agreement among judges were considered clinically validated and, therefore, applicable to the investigated context. Those with low agreement were re-evaluated and, when necessary, reformulated or excluded.

The results should be organized into summary tables, containing: ICNP^®^ term; status (validated/not validated); adjustment suggestions; and judges’ observations.

As the final product, a terminological subset of ICNP^®^ with proven clinical validity is obtained, ready for use in health record systems, teaching, or care protocols.

Considering the scope of the subset and the volume of data generated, it is recommended that the complete material be made available in a public data repository, ensuring transparency, reproducibility, and open access to information.

**Figure 1:**
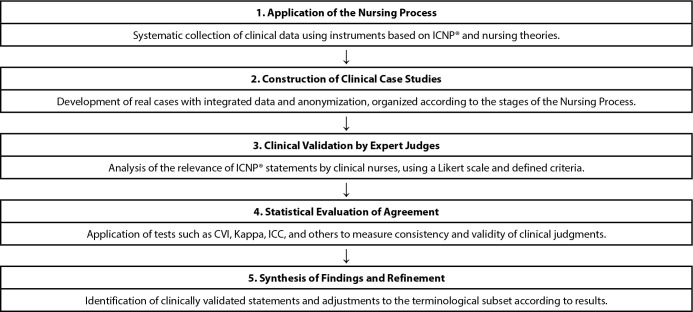
Flowchart of the stages of the clinical validation method for ICNP^®^ terminological subsets.

To facilitate understanding of the proposed methodological pathway, an illustrative flowchart is presented below, depicting the five stages that constitute the method for clinical validation of ICNP^®^ terminological subsets (Figure 1).

## DISCUSSION

The methodology proposed in this study represents a significant advancement in the clinical validation of ICNP^®^ terminological subsets, by integrating practical application, expert judgment, and statistical rigor. Unlike traditional approaches focused exclusively on content validation by experts or the Delphi technique, this method incorporates direct observation of real clinical cases, allowing for the assessment of the applicability of statements in concrete care contexts.

The operationalization of the NP as the structuring axis of clinical data collection adds robustness to the proposal, aligning records with COFEN Resolution No. 736/2024 and with nursing theories that underpin care. The construction of detailed clinical case studies, including previously validated ICNP^®^ statements and the identification of new terms, enables a holistic and contextualized analysis, which is essential for the empirical validation of subsets.

The methodological proposal was applied in research conducted in the contexts of nephrology and neonatology, demonstrating its versatility and applicability across different specialties. In the study focused on individuals with chronic kidney disease undergoing conservative treatment, the ICNP^®^ subset was clinically validated in a specialized outpatient clinic, using the Roy Adaptation Model as the theoretical framework. Real clinical cases and robust statistical indicators, such as the Intraclass Correlation Coefficient (ICC) and the Kruskal-Wallis test, were employed to confirm the relevance of the statements^(11)^. This application showed that the methodology not only validates previously defined terms but also identifies gaps and proposes new statements based on clinical practice, thereby strengthening standardized language as a health technology.

In the study conducted in a neonatal intensive care unit, the focus was the clinical validation of diagnostic indicators related to the use of Peripherally Inserted Central Catheters (PICC) in neonates. The methodological proposal enabled the evaluation of the accuracy of clinical indicators and the reliability of diagnostic inferences, using statistical tests such as ICC and Fleiss’ Kappa. The results highlighted that the practical application of ICNP^®^ in critical scenarios, such as neonatology, requires terminological refinement and rigorous empirical validation, reinforcing the relevance of the methodology presented in this article^(5)^.

The stage of clinical validation by expert judges reinforces the technical and practical nature of the proposal. By applying criteria such as clarity, relevance, and applicability, judges contribute to the refinement of statements, promoting greater adherence to care reality. The careful selection of judges, with proven experience in the thematic area and familiarity with ICNP^®^, ensures the quality of evaluations. In this regard, a methodological contribution is highlighted from a study that proposed rigorous criteria for the selection of experts in the construction of ICNP^®^ terminological subsets, considering aspects such as academic qualifications, professional experience, and involvement in clinical and academic practice^(10)^. The incorporation of these criteria strengthens the reliability of judgments and the legitimacy of the results obtained.

Regarding statistical analysis, the study proposes a coherent set of tests to measure agreement among judges, as systematized in Tables 1 and 2. Although CVI is widely used, psychometric researchers have pointed out limitations in its application. Therefore, the use of complementary tests is recommended, such as Fleiss’ Kappa, ICC, Kendall’s W, and non-parametric tests such as Kruskal-Wallis and Friedman, depending on the type of data and the objective of the analysis. This approach strengthens methodological rigor and the statistical validity of results.

The proposal also considers the inclusion of new statements, particularly interventions, provided they are clinically justified and compatible with ICNP^®^ axes. To ensure interoperability and terminological consistency, it is recommended that these new terms be developed in accordance with ISO 18104 and the SNOMED-CT ontology, which currently represent ICNP^®^ in health information systems. ISO 18104 establishes principles for structuring nursing diagnostic and intervention statements, organizing them into semantic axes that favor standardization and computational readability. SNOMED-CT, as an internationally recognized clinical ontology, enables ICNP^®^ terms to be integrated into electronic health record systems, expanding interoperability across different platforms and healthcare services.

The adoption of these standards is essential for new statements to be safely and effectively incorporated into electronic health records, clinical decision support systems, and interoperable databases, thereby enhancing the visibility of nursing practice and contributing to the quality of health information. Furthermore, such semantic compatibility facilitates data sharing among institutions, epidemiological analysis, and the development of care indicators based on standardized language.

In summary, the methodology presented in this study contributes to the strengthening of standardized nursing language by proposing a replicable, applicable model aligned with the demands of clinical practice. Its articulation between theory, practice, and statistics enables the qualification of nursing records, improvement of clinical reasoning, and expansion of the profession’s visibility within health systems.

### Study limitations

The reliance on experts for the validation stage may introduce subjective bias, especially when there is heterogeneity in the training or experience of clinical judges. The scarcity of standardized protocols for the clinical validation of ICNP^®^ hinders comparisons across studies and methodological consolidation, constituting a relevant limitation. Furthermore, it is acknowledged that the proposed methodology may require adjustments and refinements during its operationalization phase, considering the specificities of different care settings.

As a future implication, the development of national guidelines is suggested to guide the clinical validation of terminological subsets, promoting greater methodological uniformity and scientific rigor across studies.

### Contributions to Nursing, Health, and Public Policy

The methodological proposal presented contributes to the strengthening of the NP by offering a structured and replicable model for the clinical validation of ICNP^®^ terminological subsets. This model is capable of enhancing clinical reasoning, increasing patient safety, and ensuring standardized documentation.

In the field of health, the proposed methodology shows potential for integration with digital technologies, such as electronic health record systems and clinical applications that employ ICNP^®^ language. This interoperability can expand the visibility of professional practice, facilitate standardized documentation, and contribute to real-time analysis of clinical data, thereby promoting advances in care management and evidence-based nursing research.

In the realm of public policy, the proposal provides evidence supporting the incorporation of protocols based on standardized language, strengthening nurses’ autonomy and promoting safer, more effective, and user-centered care practices.

## CONCLUSIONS

The methodological proposal presented in this study proved to be innovative for the clinical validation of ICNP^®^ terminological subsets, demonstrating feasibility in hospital settings. This perspective reinforces the use of standardized nursing language as a health technology, with the potential to enhance the Nursing Process, increase patient safety, and support public policies aimed at care management.

By combining clinical case studies with statistical tests, it becomes possible to evaluate the relevance, clarity, and applicability of subsets, thereby fostering the improvement of clinical reasoning and qualifying professional decision-making.

## Data Availability

The research data are available within the article.
