# Supplementary material for: Proposal of a method for clinical validation of ICNP® terminological subsets in hospital practice
Source: Rev Bras Enferm. 2026 Jul 31;79:e20250492. doi: 10.1590/0034-7167-2025-0492 (PMC13435853; doi:10.1590/0034-7167-2025-0492)
Supplement: Translation

# Translation: Proposta de método para validação clínica de subconjuntos terminológicos da CIPE^®^ na prática hospitalar

**DOI:** 10.1590/0034-7167-2025-0492pt

**Authors:** Richardson Augusto Rosendo da Silva, Harlon França de Menezes

**Affiliations:** I Universidade Federal do Rio Grande do Norte. Natal, Rio Grande do Norte, Brasil

**Keywords:** Processo de Enfermagem, Métodos, Terminologia Padronizada em Enfermagem, Diagnóstico de Enfermagem, Pesquisa em Enfermagem Clínica.

## Abstract

**Objetivos:**

descrever uma proposta de método para validação clínica de subconjuntos terminológicos da Classificação Internacional para a Prática de Enfermagem (CIPE^®^), aplicável a cenários hospitalares.

**Métodos:**

trata-se de um estudo descritivo que propõe estratégias metodológicas para validação clínica de subconjuntos, incorporando aplicação prática em cenários reais de cuidado, avaliação por juízes clínicos e testes estatísticos de concordância.

**Resultados:**

o método é composto por cinco etapas: (1) Aplicação do Processo de Enfermagem, com foco na coleta sistematizada de dados clínicos; (2) Construção de estudos de caso clínico; (3) Validação clínica dos enunciados da CIPE^®^, realizada por juízes clínicos enfermeiros; (4) Avaliação da concordância entre juízes, utilizando-se estatística robusta; e (5) Síntese dos achados e refinamento do subconjunto terminológico.

**Conclusões:**

o método pode ser replicado em diferentes contextos clínicos, fortalecendo a cientificidade e a visibilidade da prática de enfermagem.

## INTRODUÇÃO

A consolidação do Processo de Enfermagem (PE) como método científico que orienta a prática clínica da enfermagem exige a adoção de uma linguagem padronizada, capaz de expressar com clareza os julgamentos clínicos do enfermeiro, os resultados esperados e as intervenções implementadas. Nesse contexto, a Classificação Internacional para a Prática de Enfermagem (CIPE^®^) tem se destacado como uma terminologia enumerativa, combinatória e de referência global, destinada a apoiar o registro, a comunicação e a análise do cuidado de enfermagem em diversos cenários assistenciais^(1)^.

A CIPE^®^ possibilita a construção de subconjuntos terminológicos voltados a populações específicas, ampliando a precisão e a aplicabilidade do PE em situações clínicas concretas^(2,3)^. Assim, os subconjuntos configuram-se como recursos estratégicos e tecnológicos que fortalecem a documentação do PE, ao oferecer diagnósticos, intervenções e resultados alinhados à linguagem padronizada e à prática baseada em evidências. O Conselho Internacional de Enfermeiros recomenda a criação de subconjuntos terminológicos adaptados às necessidades locais e às diferentes realidades assistenciais^(4)^.

Entretanto, a etapa de validação clínica, responsável por verificar a aplicabilidade dos subconjuntos no contexto real de cuidado, permanece pouco explorada na produção científica nacional, o que limita sua incorporação efetiva às rotinas clínicas e aos sistemas de informação em saúde^(5,6)^.

Uma revisão de escopo recente reforça a validação clínica como etapa essencial para a consolidação dos subconjuntos terminológicos da CIPE^®^ na prática assistencial. O estudo analisou 15 publicações e evidenciou carência de uniformidade metodológica, predominância de estudos de caso e concentração geográfica das pesquisas. Destacou-se, ainda, a ausência da validação clínica como fase estruturante do PE, o que demonstra a necessidade de diretrizes mais robustas. Esses achados sustentam que a validação clínica deve ocupar posição central, assegurando aplicabilidade, rigor científico e impacto na qualidade do cuidado em saúde^(6)^.

Essa lacuna aponta para a necessidade urgente de metodologias inovadoras, rigorosas e replicáveis, capazes de avaliar, com base em situações clínicas reais, a pertinência dos enunciados de enfermagem propostos. A validação clínica não apenas fortalece a segurança e a acurácia das decisões de enfermagem, como também contribui para a evolução epistemológica da linguagem profissional padronizada.

A metodologia proposta neste estudo foi desenvolvida pelo líder do Laboratório de Pesquisa em Saúde e Enfermagem no Cuidado às Pessoas em Condições Agudas e Crônicas (LAPAC) do Departamento de Enfermagem da Universidade Federal do Rio Grande do Norte (UFRN). O grupo é certificado e cadastrado no Diretório de Grupos de Pesquisa (DGP) da Plataforma Lattes do CNPq e possui mais de uma década de experiência na implementação e validação clínica da CIPE^®^ em diferentes cenários assistenciais.

Fundamentada em um percurso teórico-prático consolidado, a proposta resultou da análise crítica de teses, dissertações e artigos científicos que abordaram estratégias metodológicas de construção e validação de subconjuntos terminológicos da CIPE^®^. Esse processo permitiu ao grupo sistematizar etapas originais, coerentes e replicáveis.

Essa estrutura metodológica reflete o amadurecimento técnico e científico dos pesquisadores do LAPAC na aplicação do Processo de Enfermagem, na condução de validações clínicas com juízes especialistas e na utilização de ferramentas estatísticas robustas. Com isso, assegura-se rigor conceitual, aplicabilidade prática e aderência às diretrizes normativas, como a ISO 18104 e a Resolução COFEN nº 736/2024^(5,6)^.

Nesse sentido, propõe-se neste estudo uma metodologia inovadora de validação clínica de subconjuntos terminológicos da CIPE^®^, fundamentada na aplicação do PE em situações reais, na análise crítica de juízes clínicos e na utilização de múltiplos testes estatísticos para garantir robustez e aplicabilidade dos resultados.

O estudo apresenta uma inovação tecnológica aplicada à validação clínica de subconjuntos terminológicos da CIPE^®^, estruturada em uma proposta inédita e organizada para a utilização prática da linguagem padronizada em enfermagem.

A metodologia desenvolvida tem como diferencial a operacionalização do PE em ambiente clínico real, utilizando registros estruturados e a observação direta de casos clínicos para verificar a aplicabilidade dos enunciados diagnósticos, dos resultados esperados e das intervenções de enfermagem constantes em subconjuntos terminológicos previamente construídos e validados por especialistas.

## OBJETIVOS

Descrever uma proposta de método para validação clínica de subconjuntos terminológicos da Classificação Internacional para a Prática de Enfermagem (CIPE^®^), aplicável a cenários hospitalares.

## MÉTODOS

### Aspectos Éticos

Este estudo, de caráter descritivo, não incluiu seres humanos em sua execução e, portanto, não necessitou de apreciação pelo Comitê de Ética em Pesquisa.

### Trajetória descritiva

Trata-se de um estudo descritivo elaborado segundo as diretrizes *Standards for Quality Improvement Reporting Excellence* (SQUIRE 2.0), utilizadas como referência para assegurar padronização e qualidade nos relatos de pesquisas voltadas à melhoria da prática clínica. Dessa forma, garantem-se rigor científico, clareza narrativa e rastreabilidade metodológica em todas as etapas da investigação, conforme os itens previstos no SQUIRE.

Inicialmente, o estudo apresenta a descrição detalhada do problema e sua relevância assistencial, contextualizando a necessidade de qualificar o Processo de Enfermagem (PE) por meio da validação clínica de subconjuntos terminológicos, em conformidade com os itens 3 e 4 do checklist. A fundamentação teórica e o racional da intervenção são explicitados com base em modelos conceituais da CIPE^®^ e em evidências consolidadas, justificando as escolhas metodológicas e sustentando a eficácia da intervenção proposta (item 5).

Os objetivos são apresentados de forma clara e mensurável (item 6). O método descreve o contexto institucional da pesquisa, as características dos serviços, o perfil populacional e os recursos disponíveis, atendendo ao item 7. A intervenção é detalhada, incluindo a aplicação das etapas do PE, a elaboração de estudos de caso clínico, a identificação de diagnósticos, resultados e intervenções de enfermagem, a validação por juízes clínicos e a análise estatística subsequente, garantindo reprodutibilidade (item 8).

O estudo da intervenção e sua análise são descritos com precisão, evidenciando a abordagem adotada para determinar se os efeitos observados derivaram das atividades implementadas, em consonância com o item 9. As medidas utilizadas são apresentadas com definições operacionais, validade e confiabilidade, conforme o item 10, juntamente com a estratégia analítica quantitativa (item 11).

O manuscrito contempla, ainda, os aspectos éticos, incluindo autorização institucional e respeito aos princípios aplicáveis (item 12). Os resultados são organizados de forma sequencial, explicitando a evolução da intervenção, seus efeitos, interações com o contexto e achados emergentes (item 13). A discussão articula os resultados com o racional inicial e com a literatura nacional e internacional, compara achados com outros estudos, analisa implicações clínicas e reconhece limitações metodológicas (itens 14, 15 e 16).

Por fim, as conclusões destacam a utilidade da intervenção, sua aplicabilidade futura, o potencial de replicação em outros contextos assistenciais e recomendações para estudos subsequentes, contemplando integralmente o item 17. Dessa forma, o manuscrito demonstra aderência robusta ao SQUIRE 2.0 e oferece contribuição metodológica consistente para o aprimoramento da prática clínica em enfermagem.

O método é composto por cinco etapas:

1. Aplicação do Processo de Enfermagem, com foco na coleta sistematizada de dados por meio de exame clínico, entrevistas, escalas validadas e exames complementares;
2. Construção de estudos de caso clínico reais, a partir de registros de pacientes atendidos, contendo informações detalhadas e atualizadas sobre histórico, condição clínica e evolução do cuidado;
3. Validação clínica dos enunciados da CIPE^®^, realizada por juízes clínicos enfermeiros com experiência na área assistencial do contexto investigado, que analisam a pertinência dos diagnósticos, resultados e intervenções propostos, a partir da observação direta e da análise documental dos casos;
4. Avaliação da concordância entre juízes, utilizando estatísticas robustas, como o coeficiente Kappa e o Coeficiente de Correlação Intraclasse (CCI), para verificar a consistência das decisões clínicas;
5. Análise estatística e síntese dos achados, visando identificar enunciados clinicamente validados com base na frequência, pertinência e concordância entre os avaliadores.

Essas etapas são descritas em detalhe na seção Resultados. Ressalta-se que esta proposta apresenta maior aderência ao contexto hospitalar, cuja dinâmica de operacionalização do PE difere substancialmente daquela observada na Atenção Primária à Saúde (APS).

## RESULTADOS

### Etapa 1: Aplicação do Processo de Enfermagem com foco na coleta sistematizada de dados clínicos

Nesta etapa, o Processo de Enfermagem (PE) é aplicado junto a pacientes reais do cenário clínico selecionado. A coleta de dados deve ser conduzida por enfermeiros especialistas, que atendam aos seguintes critérios: experiência prática consistente na área, participação em grupos de pesquisa ou comissões técnico-científicas, produção acadêmica relacionada ao tema (artigos, capítulos ou trabalhos apresentados), além de envolvimento regular em eventos científicos e atividades de atualização profissional. Esses requisitos asseguram que o especialista possua conhecimento aprofundado e capacidade de análise crítica para contribuir de forma qualificada com o estudo.

O enfermeiro pode utilizar estratégias múltiplas, tais como entrevistas com o paciente e/ou família, exame físico, aplicação de escalas validadas, análise de exames laboratoriais e sinais vitais, além do uso do Subconjunto Terminológico da CIPE^®^, previamente validado quanto ao conteúdo. Os dados devem ser organizados de modo a permitir o julgamento clínico subsequente. Como sugestão metodológica, os enunciados de diagnósticos, resultados e intervenções de enfermagem identificados nos pacientes podem ser inseridos em planilhas do *Microsoft Office Excel* ^®^, em abas exclusivas correspondentes a cada paciente. Essas planilhas são enviadas para validação pelos enfermeiros especialistas, podendo incluir informações adicionais referentes a dados socioeconômicos, clínicos e observações pertinentes ao processo de inferência diagnóstica. Cada especialista julga, de forma independente, a presença ou ausência dos enunciados em cada planilha.

Diversos subconjuntos terminológicos da CIPE^®^ já dispõem de instrumentos específicos de coleta de dados, desenvolvidos por seus autores, como os voltados para pacientes com doença renal crônica, neonatologia, saúde da mulher e cuidados paliativos. Recomenda-se, sempre que possível, a utilização desses instrumentos originais, construídos com base em evidências clínicas e validados por especialistas da área, o que fortalece a coerência metodológica e a aplicabilidade dos enunciados no contexto assistencial.

Nessa etapa, sugere-se garantir consentimento ético e padronização das fontes de dados, registrar informações com clareza, em linguagem técnica, incluindo tempo de evolução, intervenções anteriores e resposta ao tratamento.

A coleta de dados é etapa inicial e estruturante do PE, conforme definido pela Resolução COFEN nº 736/2024^(7)^, que estabelece cinco fases interdependentes: Avaliação, Diagnóstico, Planejamento, Implementação e Evolução de Enfermagem. Para que essa coleta seja efetiva e clinicamente significativa, é essencial que o instrumento utilizado esteja embasado no subconjunto a ser validado e em um suporte teórico que funcione como referencial conceitual.

Além do Subconjunto Terminológico da CIPE^®^, o instrumento de coleta pode ser desenvolvido com base em diferentes teorias de enfermagem, que oferecem suporte para compreender o ser humano em sua totalidade e orientar o cuidado. A escolha da teoria deve estar alinhada ao público-alvo, ao contexto assistencial e aos objetivos do estudo. Independentemente da teoria adotada, o instrumento deve contemplar elementos organizados para subsidiar a etapa de Avaliação de Enfermagem, tais como:

- Dados de identificação e contexto social;
- Histórico de saúde e condições clínicas atuais;
- Sinais e sintomas observados;
- Aspectos emocionais, culturais e espirituais;
- Capacidades funcionais e limitações;
- Rede de apoio e ambiente físico.

Esses dados devem ser categorizados conforme os conceitos centrais da teoria escolhida e do subconjunto terminológico, permitindo análise sistemática e fundamentada. Essa coleta orientada possibilita:

- subsidiar o julgamento clínico para identificação de diagnósticos de enfermagem do subconjunto ou formulação de novos;
- definir intervenções coerentes com as necessidades identificadas ou elaborar novas;
- elencar resultados esperados com base em indicadores teóricos e clínicos, incluindo a possibilidade de propor resultados adicionais não presentes no subconjunto original.

A elaboração de novos enunciados deve seguir os padrões da ISO 18104, que define a estrutura de representações terminológicas em enfermagem, e ser compatível com a CIPE^®^, atualmente integrada à ontologia SNOMED-CT em sistemas de informação em saúde. Essa diretriz garante interoperabilidade semântica, consistência terminológica e integração com prontuários eletrônicos e tecnologias digitais em saúde^(8)^.

Destaca-se que a participação dos autores do subconjunto terminológico é fortemente recomendada durante o processo de validação clínica, mesmo quando o material já se encontra publicado e disponível em acesso aberto. Isso porque determinados aspectos conceituais, metodológicos e decisórios, como critérios de inclusão de termos, estruturação dos enunciados e delimitação de contextos clínicos, frequentemente não estão explicitados nas publicações originais, sendo compreendidos apenas por meio do relato direto dos autores. Essa interlocução contribui para assegurar a fidedignidade da aplicação do subconjunto e a coerência entre a proposta inicial e o processo de validação.

### Etapa 2: Construção de estudos de caso clínico

A elaboração dos casos clínicos fundamenta-se nos conceitos já propostos em estudos ^(6)^ e nos atributos da ISO 18104:2023, que define a estrutura e os elementos necessários para representar enunciados em sistemas de terminologia. Essa norma orienta a identificação de conceitos-chave, suas relações e contextos clínicos, constituindo base teórica adequada para compor um conjunto mínimo de dados no estudo de casos. Além disso, a elaboração dos casos clínicos considera os conceitos centrais do PE, conforme a Resolução COFEN nº 736/2024, e os eixos da CIPE^®^, garantindo coerência terminológica e aplicabilidade prática dos enunciados em validação.

Com base nos dados clínicos coletados, elabora-se um estudo de caso detalhado, representando a situação do paciente em um momento específico da internação ou atendimento. Esse estudo deve incluir: dados demográficos; histórico de saúde; condições clínicas atuais; sinais e sintomas relevantes; terapêutica em uso e evolução clínica; diagnósticos/resultados e intervenções de enfermagem do subconjunto da CIPE^®^; além da inclusão de novos enunciados identificados como necessários. O objetivo é transformar informações fragmentadas em um caso clínico integrador, que permita ao juiz clínico analisar a situação de forma holística.

Para assegurar anonimato e confidencialidade, os casos clínicos serão apresentados de forma despersonalizada, sem dados que permitam identificação direta ou indireta dos indivíduos. Cada caso será identificado apenas por um número sequencial (ex.: Caso 1, Caso 2, Caso 3), substituindo nomes e iniciais por códigos numéricos, conforme recomendações éticas nacionais e internacionais para pesquisas com dados clínicos.

Cada estudo de caso será avaliado por enfermeiros juízes, de forma independente. O segundo enfermeiro realizará a validação cruzada, verificando a consistência entre os dados apresentados, o julgamento clínico e a pertinência dos enunciados da CIPE^®^ aplicados. Em situações de discordância, será adotado o método de consenso, mediante discussão estruturada entre os juízes, até a obtenção de concordância mínima de 80%, conforme recomendações para validação de conteúdo e confiabilidade interavaliadores^(9)^.

Os estudos de caso serão elaborados pelo pesquisador responsável e equipe de especialistas, com experiência comprovada em PE e uso da CIPE^®^. Posteriormente, os casos serão revisados, garantindo consistência terminológica e fidedignidade das informações apresentadas aos juízes clínicos.

A construção de estudos de caso clínico constitui etapa essencial na metodologia de validação de subconjuntos terminológicos da CIPE^®^, pois possibilita avaliar a aplicabilidade dos enunciados em situações reais de cuidado. Conforme a Resolução COFEN nº 736/2024, o Processo de Enfermagem (PE) deve ser estruturado em cinco etapas inter-relacionadas, cíclicas e fundamentadas em suporte teórico e evidências científicas.

Sugere-se o seguinte roteiro para a elaboração dos casos clínicos:

1. Definição do público-alvo e contexto assistencial: identificar a população (ex.: pacientes com doença renal crônica, idosos em vulnerabilidade social); selecionar o cenário clínico (atenção primária, hospital, ambulatório, clínica); garantir alinhamento com o subconjunto CIPE^®^ a ser validado.
2. Coleta de informações clínicas: descrever dados clínicos por meio de prontuários, entrevistas e exame físico, elaborando um caso completo com histórico, sinais e sintomas, exames, condutas e contexto social.
3. Organização segundo as etapas do PE:
  - Avaliação de Enfermagem: dados subjetivos (entrevista) e objetivos (exame físico), incluindo resultados de escalas e exames laboratoriais;
  - Diagnóstico de Enfermagem: julgamento clínico das necessidades identificadas, utilizando linguagem padronizada da CIPE^®^;
  - Planejamento de Enfermagem: definição de prioridades, resultados esperados e prescrição de intervenções;
  - Implementação de Enfermagem: descrição das ações previstas, respeitando competências técnicas e articulando-se com a equipe multiprofissional;
  - Evolução de Enfermagem: avaliação dos resultados alcançados, revisão do plano e registro sistemático no prontuário.
4. Inserção dos enunciados CIPE^®^: selecionar diagnósticos, resultados e intervenções do subconjunto original; incluir novos enunciados identificados como necessários; relacioná-los aos dados do caso, justificando sua pertinência clínica.

### Etapa 3: Validação clínica dos enunciados da CIPE^®^ por juízes clínicos

Nesta etapa, enfermeiros com experiência prática na área temática (exemplo: oncologia, saúde da mulher, terapia intensiva, atenção primária, entre outros) atuam como juízes clínicos. São apresentados os estudos de caso acompanhados dos enunciados do subconjunto CIPE^®^ previamente selecionados.

Cada juiz avalia a pertinência dos enunciados em relação ao caso clínico utilizando uma escala do tipo Likert, variando de 1 a 4, em que 1 = não pertinente, 2 = pouco pertinente, 3 = pertinente, e 4 = muito pertinente, podendo ainda registrar comentários qualitativos. O uso dessa escala é recomendado por permitir uma avaliação mais refinada do grau de concordância entre os juízes, reduzindo a dicotomização das respostas e possibilitando análises estatísticas de concordância mais robustas. Outros itens de avaliação também podem ser incorporados nessa fase, tais como: clareza, aplicabilidade e relevância. Os ajustes semânticos, normalmente, são feitos na etapa de validação de conteúdo. Caso seja necessário, os autores poderão realizar os ajustes às novas versões da classificação.

Recomenda-se, como critérios para a seleção dos juízes clínicos, que o profissional atue na área específica de interesse do subconjunto, possua titulação mínima de especialista ou experiência comprovada, apresente disponibilidade para a etapa de validação e demonstre familiaridade com a CIPE^®^. Ademais, recomenda-se incorporar critérios específicos voltados à análise de aplicabilidade clínica, contemplando experiência prévia na operacionalização do PE com uso da CIPE^®^, participação em comissões ou grupos de pesquisa vinculados à temática e tempo mínimo de dois anos de prática clínica relacionada à clientela ou condição de saúde estudada^(10)^.

Esses parâmetros fortalecem o rigor metodológico e asseguram que os especialistas detenham competências teórico-práticas suficientes para avaliar a pertinência e aplicabilidade dos enunciados.

Orienta-se a utilização de ferramenta de validação como: formulário físico ou digital contendo os enunciados organizados por eixo (foco, julgamento, ação, meios, localização, tempo, cliente), acompanhado de instruções para julgamento clínico.

Para garantir a validade e a confiabilidade dos resultados, recomenda-se que o cálculo da amostra seja realizado com base na proporção esperada de concordância entre juízes, utilizando fórmulas específicas para estudos de concordância interavaliadores. Em geral, para o Índice de Validade de Conteúdo (IVC), sugere-se um mínimo de 5 a 10 juízes por área temática, enquanto para testes como Kappa de Fleiss ou ICC, o número de itens e o nível de precisão desejado devem orientar o dimensionamento amostral.

O tempo de coleta pode variar conforme o número de casos clínicos e a disponibilidade dos juízes, mas recomenda-se um cronograma previamente definido, com etapas de coleta, análise e validação distribuídas ao longo de 26 semanas^(6)^.

Quanto ao treinamento dos coletadores, é fundamental que os enfermeiros responsáveis pela coleta devem receber capacitação específica sobre o uso do instrumento, critérios de registro clínico, fundamentos da CIPE^®^ e etapas do PE, garantindo padronização, qualidade e fidedignidade das informações.

### Etapa 4: Avaliação estatística da concordância entre juízes

Nesta etapa, os julgamentos clínicos realizados pelos enfermeiros avaliadores acerca da pertinência dos enunciados diagnósticos, resultados e intervenções da CIPE^®^ são organizados em planilhas e submetidos à análise estatística. O objetivo é identificar o grau de consenso e/ou concordância entre os juízes, bem como verificar a significância estatística das variações observadas em suas respostas. Para esse fim, utiliza-se o Índice de Validade de Conteúdo (IVC), considerando como validados os enunciados que alcançam IVC ≥ 0,80.

Embora o IVC seja amplamente empregado em estudos de validação, especialmente na área da saúde, seu uso tem sido alvo de críticas por pesquisadores da psicometria. As principais limitações apontadas referem-se à ausência de medidas de precisão estatística, à subjetividade na definição de pontos de corte (como o tradicional ≥ 0,80) e à incapacidade de captar nuances na concordância entre avaliadores. Ademais, o IVC não considera o acaso, o que pode superestimar a validade de itens em contextos com número reduzido de juízes.

Diante dessas limitações, sugere-se também o cálculo do coeficiente Kappa e/ou do Coeficiente de Correlação Intraclasse (CCI), que permitem mensurar a concordância entre múltiplos juízes de forma mais robusta. Além disso, podem ser aplicados testes adicionais para verificar diferenças estatísticas entre as avaliações. Entre as ferramentas recomendadas para tais análises estão Excel, SPSS, R e softwares online com módulos específicos de concordância.

Portanto, em estudos que visam propor metodologias de validação clínica, é fundamental que os testes estatísticos utilizados estejam alinhados ao tipo de dado, ao número de avaliadores e ao objetivo da análise. Nesse sentido, a utilização dos Quadros 1 e 2 neste estudo contribui para a coerência metodológica, ao oferecer um repertório de testes aplicáveis e suas respectivas hipóteses estatísticas, fortalecendo o rigor científico e a validade dos resultados.

**Quadro 1:** Comparativo de testes estatísticos aplicáveis à validação clínica de subconjuntos terminológicos da CIPE^®^, Natal, Rio Grande do Norte, Brasil, 2025

Teste estatístico	O que avalia?	Quando usar?	Exemplo prático	Interpretação
**IVC** (Índice de Validade de Conteúdo)	Proporção de juízes que consideram o item pertinente	Quando as respostas são binárias (sim/não)	12 de 15 juízes validam um diagnóstico → IVC = 0,80	IVC ≥ 0,80 indica que o item é clinicamente válido
**Kappa de Fleiss**	Grau de concordância além do acaso entre 3 ou mais juízes	Quando ≥ 3 juízes avaliam a pertinência de itens (sim/não)	Três grupos de juízes avaliam 25 diagnósticos	>0,80: excelente concordância; < 0,40: fraca concordância
**ICC (CCI)**	Consistência dos escores atribuídos entre juízes	Quando se utiliza escala ordinal (ex: Likert) com ≥ 2 juízes	Juízes avaliam 10 intervenções numa escala de 1 a 4	ICC > 0,75: boa concordância; ICC > 0,90: excelente
**W de Kendall**	Grau de concordância em ranqueamento de itens por juízes	Quando juízes ranqueiam/ordenam itens em ordem de importância	Juízes ordenam 8 intervenções da mais para a menos adequada	W próximo de 1: alta concordância; W < 0,5: baixa concordância
**Teste binomial**	Se a proporção de “sim” difere de uma proporção teórica	Para verificar se a proporção de respostas esperadas (ex: 80%) é atingida	Espera-se 80% de concordância, mas apenas 9/15 juízes validam o item	p < 0,05 indica diferença significativa da proporção esperada
**Teste de Kruskal-Wallis**	Diferença entre as medianas de ≥ 3 grupos independentes	Quando juízes são de diferentes áreas ou perfis	Juízes de cenários assistenciais avaliam a pertinência de um conjunto de diagnósticos	p < 0,05: pelo menos um grupo difere; usar post-hoc (Dunn) para localizar a diferença
**Friedman**	Diferença nas avaliações repetidas de ≥ 3 condições pelos mesmos juízes	Quando os mesmos juízes avaliam múltiplos itens em escala ordinal	Cinco juízes avaliam 6 diagnósticos de 1 a 4	p < 0,05: diferença significativa entre os itens; pode-se usar post-hoc de Dunn
**ANOVA de medidas repetidas**	Diferença nas médias de avaliações feitas pelos mesmos juízes	Quando há ≥ 3 avaliações de um mesmo grupo com dados paramétricos	Mesmos juízes avaliam 4 diagnósticos em momentos distintos	p < 0,05: existe diferença entre médias; post-hoc (Tukey ou Bonferroni) identifica onde está a diferença
**Wilcoxon**	Diferença entre duas avaliações pareadas (não paramétrica)	Quando os mesmos juízes avaliam os itens em duas rodadas	Comparação entre avaliação 1 e 2 após ajuste de termos	p < 0,05: houve mudança significativa nas avaliações
**t-Student pareado**	Diferença entre médias de duas avaliações pareadas com dados normais	Quando as duas rodadas de avaliação têm distribuição normal	Mesmos juízes avaliam 20 termos antes e depois da revisão	p < 0,05: as médias são significativamente diferentes entre os dois momentos

**Quadro 2:** Hipóteses estatísticas adequadas para cada teste aplicado na validação clínica de subconjuntos terminológicos da CIPE^®^, Natal, Rio Grande do Norte, Brasil, 2025

Teste	H₀ (Hipótese nula)	H₁ (Hipótese alternativa)
IVC	(IVC ≤ 0,80 não valida o item)	(IVC ≥ 0,80 valida o item)
Kappa de Fleiss	A concordância entre juízes é igual ao acaso	A concordância é maior que o acaso
ICC	Não há consistência entre os juízes (ICC ≈ 0)	Há consistência estatisticamente significativa (ICC > 0)
Kendall W	Não há concordância entre os juízes (W ≈ 0)	Há concordância significativa (W > 0)
Kruskal-Wallis	As medianas dos grupos são iguais	Pelo menos uma mediana é diferente
ANOVA de medidas repetidas	As médias das avaliações são iguais	Pelo menos uma média é diferente
Friedman	As distribuições das avaliações são iguais	Pelo menos uma é diferente
Wilcoxon	As distribuições entre as rodadas são iguais	As distribuições são diferentes
t-Student pareado	As médias são iguais entre duas rodadas	As médias são diferentes
Teste binomial	A proporção observada = proporção esperada (exemplo: 0,80)	A proporção observada ≠ proporção esperada

A proposta metodológica delineada busca garantir coerência entre os objetivos da validação clínica de subconjuntos terminológicos da CIPE^®^ e os procedimentos estatísticos adotados. Para isso, os Quadros 1 e 2 foram elaborados com o intuito de orientar, de forma sistematizada, a escolha dos testes estatísticos mais adequados conforme o tipo de dado, o número de juízes e o objetivo específico da análise.

O Quadro 1 apresenta um comparativo entre diferentes testes de concordância e validade, destacando suas aplicações, exemplos práticos e critérios de interpretação. Já o Quadro 2 explicita as hipóteses estatísticas (nula e alternativa) associadas a cada teste, reforçando a fundamentação teórica e metodológica da análise.

Dessa forma, o uso desses quadros assegura alinhamento entre a natureza do estudo de validação clínica e os métodos estatísticos empregados, promovendo maior rigor, transparência e reprodutibilidade dos resultados.

O Quadro 2 apresenta as hipóteses estatísticas (nula e alternativa) adequadas para cada teste aplicado na validação clínica de subconjuntos terminológicos da CIPE^®^.

### Etapa 5: Síntese dos achados e refinamento dos subconjuntos terminológicos

Os enunciados que apresentaram alta concordância entre os juízes foram considerados validados clinicamente e, portanto, aplicáveis ao contexto investigado. Aqueles com baixa concordância foram reavaliados e, quando necessário, reformulados ou excluídos.

Os resultados devem ser organizados em quadros-síntese, contendo: termo da CIPE^®^; status (validado/não validado); sugestões de ajustes; observações dos juízes.

Como produto final, obtém-se um subconjunto terminológico da CIPE^®^ com validade clínica comprovada, pronto para utilização em sistemas de registro, ensino ou protocolos assistenciais.

Considerando a extensão do subconjunto e o volume de dados gerados, sugere-se que o material completo seja disponibilizado em repositório público de dados, assegurando transparência, reprodutibilidade e acesso aberto às informações.

Para facilitar a compreensão do percurso metodológico proposto, apresenta-se a seguir um fluxograma ilustrativo com as cinco etapas que constituem o método de validação clínica de subconjuntos terminológicos da CIPE^®^ (Figura 1).

**Figura 1:** Fluxograma das etapas do método de validação clínica de subconjuntos terminológicos da CIPE®

## DISCUSSÃO

A metodologia proposta neste estudo representa um avanço significativo na validação clínica de subconjuntos terminológicos da CIPE^®^, ao integrar aplicação prática, julgamento especializado e rigor estatístico. Diferentemente de abordagens tradicionais centradas exclusivamente na validação de conteúdo por especialistas ou na técnica Delphi, este método incorpora a observação direta de casos clínicos reais, permitindo avaliar a aplicabilidade dos enunciados em contextos assistenciais concretos.

A operacionalização do Processo de Enfermagem (PE) como eixo estruturante da coleta de dados clínicos confere robustez à proposta, ao alinhar os registros à Resolução COFEN nº 736/2024 e às teorias de enfermagem que fundamentam o cuidado. A construção de estudos de caso clínico detalhados, com inserção de enunciados da CIPE^®^ previamente validados e identificação de novos termos, favorece uma análise holística e contextualizada, essencial para a validação empírica dos subconjuntos.

A proposta metodológica foi aplicada em pesquisas desenvolvidas nos contextos da nefrologia e da neonatologia, evidenciando sua versatilidade e aplicabilidade em diferentes especialidades. No estudo voltado para pessoas com doença renal crônica em tratamento conservador, o subconjunto CIPE^®^ foi validado clinicamente em um ambulatório especializado, tendo como referencial teórico o Modelo de Adaptação de Roy. Foram utilizados casos clínicos reais e indicadores estatísticos robustos, como o Coeficiente de Correlação Intraclasse (ICC) e o teste de Kruskal-Wallis, para confirmar a pertinência dos enunciados^(11)^. Essa aplicação demonstrou que a metodologia permite não apenas validar termos previamente definidos, mas também identificar lacunas e propor novos enunciados com base na prática clínica, fortalecendo a linguagem padronizada como tecnologia em saúde.

Já no estudo realizado em uma unidade de terapia intensiva neonatal, o foco foi a validação clínica de indicadores diagnósticos relacionados ao uso do Cateter Central de Inserção Periférica (CCIP) em neonatos. A proposta metodológica possibilitou avaliar a acurácia dos indicadores clínicos e a confiabilidade das inferências diagnósticas, utilizando testes estatísticos como o ICC e o Kappa de Fleiss. Os resultados evidenciaram que a aplicação prática da CIPE^®^ em cenários críticos, como a neonatologia, exige refinamento terminológico e validação empírica rigorosa, reforçando a relevância da metodologia apresentada neste artigo^(5)^.

A etapa de validação clínica por juízes especializados reforça o caráter técnico e prático da proposta. Ao utilizar critérios como clareza, pertinência e aplicabilidade, os juízes contribuem para o refinamento dos enunciados, promovendo maior aderência à realidade assistencial. A seleção criteriosa dos juízes, com experiência comprovada na área temática e familiaridade com a CIPE^®^, garante a qualidade das avaliações. Nesse sentido, destaca-se a contribuição metodológica de estudo que propôs critérios rigorosos para a seleção de especialistas na construção de subconjuntos terminológicos da CIPE^®^, considerando aspectos como titulação, experiência profissional e envolvimento com a prática clínica e acadêmica^(10)^. A incorporação desses critérios fortalece a confiabilidade dos julgamentos e a legitimidade dos resultados obtidos.

No que se refere à análise estatística, o estudo propõe um conjunto coerente de testes para mensurar a concordância entre juízes, conforme sistematizado nos Quadros 1 e 2. Embora o IVC seja amplamente utilizado, reconhece-se que pesquisadores da psicometria têm apontado limitações em sua aplicação. Por isso, recomenda-se a utilização de testes complementares, como o coeficiente Kappa de Fleiss, o ICC, o W de Kendall e testes não paramétricos como Kruskal-Wallis e Friedman, conforme o tipo de dado e o objetivo da análise. Essa abordagem fortalece o rigor metodológico e a validade estatística dos resultados.

A proposta também contempla a possibilidade de inclusão de novos enunciados, especialmente intervenções, desde que justificados clinicamente e compatíveis com os eixos da CIPE^®^. Para garantir interoperabilidade e consistência terminológica, recomenda-se que esses novos termos sejam elaborados conforme a norma ISO 18104 e a ontologia SNOMED-CT, que atualmente representam a CIPE^®^ em sistemas de informação em saúde. A norma ISO 18104 estabelece princípios para a estruturação de enunciados diagnósticos e de intervenções de enfermagem, organizando-os em eixos semânticos que favorecem a padronização e a legibilidade computacional. Já a SNOMED-CT, como ontologia clínica internacionalmente reconhecida, permite que os termos da CIPE^®^ sejam integrados a sistemas eletrônicos de registro, ampliando a interoperabilidade entre diferentes plataformas e serviços de saúde.

A adoção desses padrões é essencial para que os novos enunciados possam ser incorporados de forma segura e eficaz em prontuários eletrônicos, sistemas de apoio à decisão clínica e bases de dados interoperáveis, promovendo maior visibilidade da prática de enfermagem e contribuindo para a qualidade da informação em saúde. Além disso, essa compatibilidade semântica facilita o compartilhamento de dados entre instituições, a análise epidemiológica e a construção de indicadores assistenciais baseados em linguagem padronizada.

Em síntese, a metodologia apresentada neste estudo contribui para o fortalecimento da linguagem padronizada na enfermagem, ao propor um modelo replicável, aplicável e alinhado às exigências da prática clínica. Sua articulação entre teoria, prática e estatística permite qualificar os registros de enfermagem, aprimorar o raciocínio clínico e ampliar a visibilidade da profissão nos sistemas de saúde.

### Limitações do estudo

A dependência de especialistas para a etapa de validação pode introduzir viés subjetivo, especialmente quando há heterogeneidade na formação ou na experiência dos juízes clínicos. A escassez de protocolos padronizados para validação clínica da CIPE^®^ dificulta comparações entre estudos e a consolidação metodológica, configurando-se como uma limitação relevante. Ademais, reconhece-se que a proposta apresentada poderá demandar ajustes e aprimoramentos em sua fase de operacionalização, considerando as especificidades dos diferentes cenários assistenciais.

Como implicação futura, sugere-se o desenvolvimento de diretrizes nacionais que orientem a validação clínica de subconjuntos terminológicos, promovendo maior uniformidade metodológica e rigor científico entre as produções.

### Contribuições para a área da enfermagem, saúde ou políticas públicas

A proposta metodológica apresentada contribui para o fortalecimento do Processo de Enfermagem, ao oferecer um modelo estruturado e replicável de validação clínica de subconjuntos terminológicos da CIPE^®^. Esse modelo é capaz de qualificar o raciocínio clínico, ampliar a segurança do paciente e garantir registros padronizados.

No campo da saúde, a metodologia proposta apresenta potencial para integração com tecnologias digitais, como sistemas de prontuário eletrônico e aplicativos clínicos que utilizem linguagem CIPE^®^. Essa interoperabilidade pode ampliar a visibilidade da prática profissional, facilitar o registro padronizado e contribuir para a análise de dados clínicos em tempo real, promovendo avanços na gestão do cuidado e na pesquisa em enfermagem baseada em evidências.

No âmbito das políticas públicas, a proposta oferece evidências que apoiam a incorporação de protocolos baseados em linguagem padronizada, fortalecendo a autonomia do enfermeiro e promovendo práticas assistenciais mais seguras, resolutivas e centradas no usuário.

## CONCLUSÕES

A proposta metodológica apresentada neste estudo mostrou-se inédita para a validação clínica de subconjuntos terminológicos da CIPE^®^, demonstrando viabilidade em ambientes hospitalares. Essa perspectiva reforça a utilização da linguagem padronizada em enfermagem como uma tecnologia em saúde, com potencial para aprimorar o Processo de Enfermagem (PE), ampliar a segurança do paciente e apoiar políticas públicas voltadas à gestão do cuidado.

Ao combinar estudos de caso clínico com testes estatísticos, torna-se possível avaliar a relevância, a clareza e a aplicabilidade dos subconjuntos, favorecendo o aprimoramento do raciocínio clínico e qualificando a tomada de decisão profissional.

## Data Availability

Os dados de pesquisa estão disponíveis no corpo do artigo.
